# Regional inequalities in benzene exposures across the European petrochemical industry: A Bayesian multilevel modelling approach

**DOI:** 10.1016/j.envint.2019.05.006

**Published:** 2019-11

**Authors:** Calvin Jephcote, Alice Mah

**Affiliations:** Department of Sociology, University of Warwick, Coventry CV4 7AL, United Kingdom

**Keywords:** Bayesian multilevel modelling, Environmental justice, Europe, Mortality rates, Petrochemical industry, Polluting practices

## Abstract

**Background:**

Pollutants released from the petrochemical industry are thought to increase the risk of mortality in fence-line communities, yet the results from previous studies are often inconsistent and lack a global perspective, hampered by the absence of cohesive cross-country research.

**Objectives:**

To provide the first Pan-European analysis of benzene exposures from the petrochemical industry, connecting polluting practices to pollution episodes and disparities in regional mortality rates, identifying the measures of best environmental practice to mitigate adverse outcomes.

**Methods:**

The activity, classification and location of onshore petrochemical facilities within EU-28 Member States were extracted from the ‘European Pollutant Release and Transfer Register’ (E-PRTR), which holds records on 31,753 industrial operations for the reporting period of 2007–15. Parent company records were collected from the Moody's Analytics Amadeus database of 487,338 active companies across Europe. The EUROSTAT census provided records of income, life expectancy, and the underlying demographics used to calculate standardised health outcomes based on 9,936 sub-populations within the NUTS2 regions. The European Environment Agency provided ambient concentrations of benzene from 579 air quality stations. Bayesian multilevel models were constructed to account for variability caused by spatial hierarchical structures, uncertainty in the estimates, and to incorporate both individual and group-level influences.

**Results:**

Higher levels of benzene emissions from petrochemical operations, both overall and in terms of specific pollution events, were associated with increased mortality rates for nearby residential populations, particularly in areas with socioeconomic deprivation. We identify uneven patterns of polluting practices within the industry, and locations that require epidemiological studies.

**Conclusions:**

While petrochemical facilities in all European Union regions are regulated to be compliant with the annual average benzene limit of 5 μg/m^3^, uneven exposures still present regional health inequalities. We recommend extending benzene regulations to an hourly or daily limit, alongside the strengthening of regulation for other toxic petrochemical releases.

## Highlights

• A continental analysis of benzene exposures from the petrochemical industry
• Connects polluting practices to pollution episodes and regional health disparities
• Identifies uneven patterns of polluting practices within the industry
• Locates places that require further epidemiological research
• Recommends extending benzene regulations to an hourly or daily limit

## 1 Introduction

The European Union is recognized as a global leader in environmental and health regulations, informed by its precautionary principle to risk. Despite these regulations, approximately 53% of European citizens are currently exposed to air pollutant levels that exceed air quality standards set by the World Health Organisation, and it is estimated that air pollution is accountable for anywhere between 78,000 to 428,000 premature deaths across Europe per annum (EEA, 2017).

While these estimates are attributed to exposures of either nitrogen or particulates, frequently measured because of their release quantities and often visible health symptoms, it is near impossible to apportion blame to one component from a concoction of respirable pollutants. Identifying the specific source of anthropogenic particulates and nitrogen dioxide, which may originate from industrial and mobile forms of combustion, is equally challenging. Sulphur dioxide is traditionally used to distinguish the contributions of general industry, with its historical legacy concerning pollution episodes, the pollutant is still measured at 36% of the 3,316 monitoring stations across Europe in 2017, despite emissions falling by up to 97% since 1970 (EEA, 2018; DEFRA, 2018). Still, difficulties often remain in identifying a tracer pollutant to act as a proxy for the broader impact of an individual industrial sector. Releases unique to the petrochemical industry include the BTEX group of volatile organic compounds (benzene, toluene, ethylbenzene, and xylene), which are classified under various levels of carcinogenicity. Of these, benzene is the most widely reported – 13% of Europe's monitoring network (EEA, 2018).

Benzene is a natural component of crude petroleum at levels up to 4 g/l and is one of the elementary petrochemicals used to chemically synthesise new products, which may contain up to 15% volume benzene (WHO, 2010b; IARC, 1989). This genotoxic carcinogen predominantly exists in the vapour phase, with residence times varying between one day and two weeks (WHO, 2010a).

Occupational exposure studies have identified a series of adverse haematological effects in employees that are regularly exposed to ‘low’ concentrations of benzene. Lan et al. (2004) reported a 15% reduction in the number of white blood cells following daily exposures to <1 ppm (< 3,250 μg/m^3^) over a 1-month period. The same occupational exposure levels over a 45-year period are associated with a 260% increase in the number of leukaemia mortalities (Paxton et al., 1994). National employment cohorts have since shown that employees of the upstream petroleum industry that are regularly exposed to benzene, have a 90% higher risk of developing haematological neoplasms than the general populace (Kirkeleit et al., 2008). Cancer biomarkers have even been found from daily benzene exposures < 0.1 ppm (< 325 μg/m^3^), questioning whether a safe threshold exists in relation to benzene (Kang et al., 2005; Hu et al., 2006). The World Health Organisation guidelines state that there are no safe levels of exposure to benzene, with the excess lifetime risk of leukaemia increasing by 1 in 6,000,000 for each 1 μg/m^3^ increase in airborne concentrations (WHO, 2010a).

There is a growing, yet still largely inconclusive evidence base documenting the risk of cancer among residential populations living near to petrochemical operations. An early investigation of refinery sites across Great Britain, by Wilkinson et al. (1999), found no association between residential proximity and incidence of leukaemia or non-Hodgkin's lymphoma, during 1974–1991. In contrast, Knox (1994) identified the number of childhood leukaemia incidences to increase by 26% if residing within 5 km of an oil refinery, or 25% for those within 1.25 km of a downstream facility. While the debate has stifled in the UK, interest has peaked in recent years across other European nations, particularly in France and Italy (Pasetto et al., 2012; Pascal et al., 2013; Bentayeb et al., 2015; Fazzo et al., 2016). A 25-year study of 20,327 French residents, identified an 8% increase in mortalities per 1 μg/m^3^ increase in the annual average concentration of benzene, after adjusting for pollutants from non-industrial sources (Bentayeb et al., 2015). Meanwhile, a Sicilian cohort of 7,147 petrochemical employees identified a 111% disparity in lung cancer mortality rates between manual and office workers, with the level of risk elevated by a further 71% for employees residing nearby (Pasetto et al., 2012).

Still, the debate remains largely disjointed with few studies using a consistent approach to measure petrochemical exposure, or to control for lifestyle and socioeconomic influences. Lin et al.'s (2017) meta-analysis provides the first attempt to pool some of this knowledge base, identifying no significant increase in the rate of lung cancer mortalities along fence-line communities. A revised meta-analysis found that individuals living near petrochemical facilities have a 19% higher risk of developing lung cancer (Lin et al., 2018). The World Health Organisation's semi-systematic review of the petrochemical industry indicates that inadequacies in the evidence base, may in part be addressed with uniform research at a global or continental scale, which provides a framework and set of outcomes that existing and future case studies may be compared to (WHO, 2014).

European countries abide to one of the most developed and influential bodies of environmental law, where enforcement at a continental scale has uniquely facilitated cross-country cooperation to complex issues. The right to breathe clean air was first legislated by Directive 1980/779/EEC, with the latest revision Directive 2008/50/EC establishing an annual average legal limit for benzene at 5 μg/m^3^. Following the 1953 formation of EUROSTAT there has been a consensus of “data democracy” across Europe, with recent legislation paying attention to the harmonisation and accessibility of spatial statistics (European Commission, 2003; European Commission, 2007).

This article provides the first Pan-European analysis of the petrochemical industry, connecting polluting practice to disparities in regional mortality rates, and identifying measures of corporate and regulatory practice that may be implemented to mitigate such adverse outcomes. In following this approach, the research addresses several shortcomings of the existing literature, addressing the need for comparative research at a continental scale to understand the underlying environmental issues, observe overarching trends, and to inform where future epidemiological case-studies are needed.

## 2 Materials & methods

### 2.1 Data collection

#### 2.1.1 Petrochemical facilities

Fossil fuels and petrochemicals are links in a long production chain, which involves the ‘upstream’ processing of natural gas and crude oil, ‘midstream’ transportation and the ‘downstream’ production of commercially marketable products. The financial and safety concerns of transportation are often mitigated by integrating refinery and manufacturing operations, or through the establishment of chemical complexes that adjacently house upstream and downstream processes. As a result, it is difficult to disentangle the petrochemical phase of the supply chain, let alone identify what consists as product of the petrochemical industry, considering that petrochemicals in some part form a host of essential everyday commodities.

To navigate these difficulties a bottom-up approach was adopted, in which a facilities involvement in the petrochemical industry was identified through releases of the tracer pollutant, benzene. Through capturing the extraction and manufacturing processes, the following analysis allows for a cradle-to-gate assessment of the European petrochemical industry.

The European Pollutant Release and Transfer Register (E-PRTR) contains facility-by-facility information on the release and transfers of 91 key pollutants, covered by Regulation No 166/2006 (EC, 2006). The open-access electronic database (version-12) currently holds information on 388,661 forms of pollutant releases across 31,753 industrial operations, located within EU-28 Member States for the reporting period of 2007–15.

Petrochemical facilities were identified through a series of logical steps:

a) BTEX compounds (benzene, toluene, ethylbenzene, and xylene) are tracers of the petrochemical industry. The E-PRTR contains 695 facilities with BTEX releases to the atmosphere. Toluene, ethylbenzene or xylene emissions data is recorded by fewer than 15 of these facilities. Benzene is the only aromatic hydrocarbon to be universally reported, it is a known carcinogen and therefore acts as a proxy of the petrochemical industry's wider environmental impact.
b) European industry standard classification codes were checked to ensure that the activity of each facility, corresponds to the petrochemical industry (n = 203). Upstream processes include the manufacture of refined petroleum products (NACE 19.20, 23.20) and gases for industrial purposes (NACE 20.11, 35.21, 40.21). Commercial downstream processes concern the manufacturing of organic chemicals (NACE 20.14, 20.59, 24.00, 24.10, 24.14, 24.60, 24.66), dyes (NACE 24.12), man-made fibres (NACE 20.60), primary plastics (NACE 20.16, 24.16, 25.22), pesticides (NACE 24.20), or basic pharmaceuticals (NACE 21.10, 24.41, 24.42).
c) E-PRTR sector codes describing the main industrial activity of each facility, were used to remove any remaining anomalies (n = 189): The manufacture of inorganic chemicals and synthetic graphite (EPER 4.2/4.3/9.3/9.4), off-site waste water treatment (EPER 5.7), or thermal power stations and other combustion installations (EPER 1.1/1.3)
d) Records were merged if the coordinates associated with the centre point of two or more ‘unique’ facilities from the E-PRTR database were <50 m apart (n = 59). These merges were validated visually using satellite imagery and through records of ownership, to form a dataset of 157 petrochemical facilities.

**Fig. 1:**
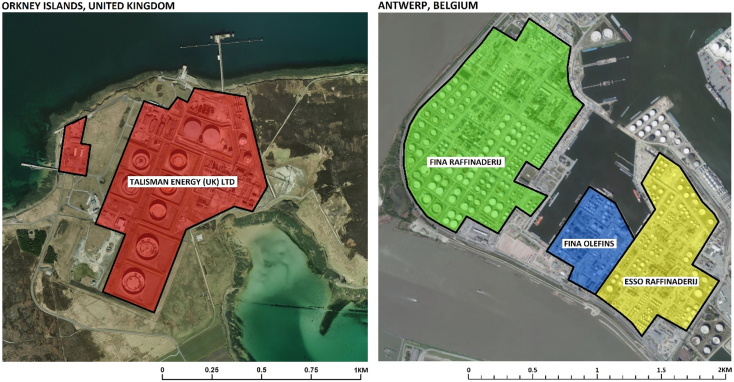
Satellite informed digitisation of petrochemical facility footprints (Source: ESRI, DigitalGlobe).

The centroid coordinates of each facility were verified against satellite imagery and online records, with the spatial extent of each facility digitised through a geographic information system (Fig. 1). Severe geographical errors were present in 13% of the facility records, defined as a difference of >2 km between the true centroid coordinates and those provided by E-PRTR reports. Any geographical differences were corrected to prevent the misidentification of petrochemical operations. A single off-shore location was removed (n = 156).

In total, 45% of the facilities contained monitoring equipment that specifically measured benzene emission leaving the site. A further 43% of facilities, were calculated using chemical mass balances based on the measurement of material entering and leaving the operation (i.e. assisted by the measurement of other pollutants). Only 12% of the petrochemical facilities provide estimations of their benzene releases. The use of estimated data is random, geographically (9% of western, 15% of northern and 7% of southern European facilities) and by petrochemical sector (8% of upstream and 3% of downstream facilities).

Satellite imagery was used to identify structures that had been constructed for petrochemical activity, to determine the size of each facility. These facility footprints were used as a proxy of activity, because site-specific information on the volume of processed petroleum products is not publicly available. A classification scheme based on a two-fold increase in facility size, was found to best represent the relationship between potential activity and emission releases (Spearman's Rho = 0.75 [P < 0.01]). Table 1 summarises the relationship between facility size (the proxy of site activity) and an incremental increase in benzene emissions.

**Table 1:** Distribution of annually recorded benzene emissions (tonnes per year) by petrochemical facility size, where “Small” is <0.5km^2^, “Medium” = 0.5-1 km^2^, “Large” = 1–2 km^2^, and “Extra-Large” >2km^2^.

Distribution	Facility Extent				Total (n=156)
	Small (N=56)	Medium (N=45)	Large (N=45)	Extra-large (N=10)	
**25**^**th**^**Percentile**	1.6 t/y	3.8 t/y	12.4 t/y	86.1 t/y	3.2 t/y
**50**^**th**^**Percentile**	2.6 t/y	7.4 t/y	19.3 t/y	124.6 t/y	7.9 t/y
**75**^**th**^**Percentile**	4.7 t/y	13.3 t/y	28.0 t/y	198.9 t/y	22.6 t/y

Information relating to the corporate structure and finances of the parent companies operating these facilities were collected from the Moody's Analytics Amadeus database, which contains records on 487,338 active companies across Europe. The 156 petrochemical facilities were associated with 111 unique parent company records, although some of these are subsidiaries of a larger corporation (for example, BP Chembel, BP Chemicals Ltd., and BP Oil UK Ltd). The size of, and degree of independence between a company and its shareholders in 2015 were directly acquired from the database. Annual records for the period 2011–15, were used to calculate 5-year average measures of assets per employee, profit margins and solvency. Missing asset (n = 7), profit (n = 9) and solvency (n = 2) records were supplemented with data from preceding years, or manually calculated from underlying information. Parent companies with missing information were often supplemented with values from the lower or upper quartiles (solvency = 100%; profit = 89%; asset = 86%) of said measures – the extremes.

Several open access datasets were used to calculate a series of proximity metrics for each facility. Proximity to a port location was derived from the National Geospatial-Intelligence Agency World Port Index (WPI), which contains coordinates for 3,669 major ports and terminals worldwide. Settlement proximity and exposure counts were calculated from the 1x1km gridded population datasets, accessed via the Eurostat and European Forum for Geography and Statistics GEOSTAT initiative. Proximity to an urban cluster was used to define whether a petrochemical facility was in an urban (<0.5 km), peri-urban (0.5-5 km) or rural (>5 km) location. GEOSTAT define an urban cluster as a collection of adjacent grid cells each containing >300 inhabitants per km^2^, which form a settlement of >5,000 persons. Residents within a 1 km catchment area formed an exposed population count for each facility, in accordance to cited disparities in respiratory outcomes near petrochemical operations (Smargiassi et al., 2009, Simonsen et al., 2010).

#### 2.1.2 Regional records

Eurostat is a Directorate-General of the European Commission, responsible for the harmonisation of regional information across member states and candidates for accession, in accordance to Regulation (EC) No 1059/2003 and Directive 2007/2/EC. The electronic census is open-access and contains statistical information in accordance to the Nomenclature of Territorial Units for Statistics (NUTS) geoclassification, which hierarchically divides EU-28 Member States countries into 98 macro-regions (NUTS1), 276 regions (NUTS2) and 1,342 micro-regions (NUTS3).

For consistency, all data was collected at a regional level (NUTS2), which is the highest resolution for records on the absolute number of deaths by cause and hospital discharges by diagnosis. Standardised rates of malignant neoplasm and all causes of mortality were calculated using the records of 9,936 gender-by-age-by-region subpopulations. This procedure removes the confounding influence of gender and age-related risks, then standardises the adjusted-rates in accordance to an expected population structure, facilitating the direct comparison of NUTS2 communities (see Appendix 1). To account for temporal fluctuations, these annual average mortality rates (per 100,000 persons) were calculated for the 10-year period of 2006–15. Hospital discharge records were found to only be reported by 9 of the EU-28 Member States, preventing its use in any further analysis.

Regional records for years of life expectancy and Gross domestic product in purchasing power standards (GDP-PPS) were collected for the 10-year period of 2006–15. GDP-PPs provides a measurements of a region's economic growth in euros, which is adjusted to account for price disparities between countries. A comparison of 2006 and 2015 data reveals that trends in life expectancy (R^2^ = 0.98) and GDP-PPS (R^2^ = 0.92) have remained temporally stable.

Maximum hourly air quality measurements of Benzene in the period 2013–15, were accessed from the Airbase open-access service provided by the European Environment Agency (EEA, 2018). EU-28 Member States are bound under Decision 97/101/EC to engage in the reciprocal exchange of ambient air quality information. Across Europe, 579 air quality stations were identified to provide valid hourly benzene concentration capture rates of at least 50% over the 3-year period. Cross-country ratified air pollution data was not available for the period prior to 2013. The station with the highest recorded measurement was used to represent the maximum hourly benzene concentration. In total, 118 NUTS2 regions provided suitable benzene measurements – these contained 93 petrochemical facilities, which were typically located within 4 km of a monitoring station. Annual average benzene concentrations were also collected for 209 of the NUTS regions.

Part of the analysis explores the confounding influence of other benzene sources, in the form of road-transport, which is the predominant source of pollutants in the post-industrial cityscape. The Eurostat database was used to acquire regional information on vehicle stock, and the total distances covered by various forms of road-transport. The total number of vehicle kilometres travelled in each country for buses, cars and HGVs, were allocated to the regions based on vehicle counts. Regional vehicle kilometres were further allocated based on vehicle age (Euro 0–6 classification), and fuel splits for passenger vehicles (diesel, petrol, LPG) obtained from the European Automobile Manufacturers Association. The resulting dataset was then combined with appropriate emission factors (g/km) for non-methane volatile organic compounds (NMVOCs), provided by the EEA 2016 emission inventory.

### 2.2 Bayesian multilevel modelling

The following Pan-European analysis uses multilevel models to explore three distinct yet closely related topics, with the intention of:

A. Understanding the characteristics and practices, which determine the magnitude of toxic pollutants released from individual petrochemical facilities.
B. Exploring the connection between benzene pollution events and regional (NUTS2) emissions from the petrochemical industry.
C. Establishing regional (NUTS2) relationships between the petrochemical industry and a “triple jeopardy” of social, environmental and health inequalities.

Multilevel regression models are a class of statistical models developed for the analysis of data with nested sources of variability (i.e. hierarchical structures). When researchers apply standard statistical techniques to multilevel data, the assumption of independent errors is violated. Furthermore, the multilevel model provides a coherent model that simultaneously incorporates both individual (i.e. site-specific measurements) and group-level influences (i.e. secondary information).

A Bayesian framework was preferred for the linear multilevel model analysis, following recent criticisms of frequentist or null hypothesis significance testing (Trafimow and Marks, 2015). Under a Bayesian approach, the dataset is directly used to construct likelihood functions which assign probability to the occurrence of any event. Direct inferences are formed on the parameters which are described probabilistically, allowing for a more robust uncertainty analysis of the estimates. In contrast, frequentist methods fix the models parameters and repeatedly sample the dataset to subjectively determine rejection of the null hypothesis.

Bayesian multilevel models using Markov Chain Monte Carlo (MCMC) simulation were built in the ‘runjags 2.0.4–2’ [R] package (Denwood, 2016), which interfaces to JAGS version 4.2.0 (Plummer, 2003). All models implemented a 4-chain simulation procedure, with the first 10,000 simulations removed as part of the model burn-in period, and the subsequent 10,000 sampled simulations kept for model interpretation. Coefficients are provided as the mean value of the parameter's posterior distribution, which is comparable to frequentist modelling approaches. In addition, coefficients are provided at the 95% highest density intervals (HDIs), representing the range of the posterior distribution that has a 0.95 probability of containing the true value of the parameter.

All Pan-European multilevel models follow a two-level hierarchical nesting structure:

• The ‘pollutant prediction’ models of section 1, were based on measurements at 130 petrochemical facilities (level-1) clustered within 60 NUTS2 regions (level-2).
• The ‘peak pollution’ models of section 2, evaluates the maximum 1-hour benzene concentrations from 130 NUTS2 regions (level-1) clustered within 28 European countries (level-2).
• The ‘triple-jeopardy’ models of section 3, were constructed from measurements across 269 NUTS2 regions (level-1) clustered within 28 European countries (level-2).

For example, a model exploring the relationship between emission rates and the characteristics of petrochemical facilities was reported as:

$$y_{\mathrm{ij}}\sim \mathrm{Normdist}\;\left(\;\alpha_{j}+\left[\beta_{0}+\beta_{1}\;{\mathrm{IndustrialSector}}_{\mathrm{ij}}+\ldots +\beta_{n}\;{\mathrm{Ownership}}_{\mathrm{ij}}\right],\sigma_{y}^{2}\;\right)$$

$$\alpha_{j}\sim \mathrm{Normdist}\;\left(\;{\delta_{10}}_{j}+\delta_{11}\;{\mathrm{RegionalGDP}}_{j},\sigma_{\alpha }^{2}\;\right)$$

where ***y***_***ij***_ is the rate of benzene emission released at facility ***i***, which is geographically positioned within region ***j*** (level-2). The intercept (i.e. facility emission baseline) is ***β***_**0**_, with the contribution of any other variables captured by fixed effect estimates ***β***_**1**_***,*** … ***,β***_***n***_.

A second intercept ***δ***_**10**_ defines the contribution of geographical cluster ***j***, which contains several facilities. The influence of group-level predictors from secondary data sources may be explained further by the inclusion of additional level-2 coefficients ***δ*** within ***α*** (i.e. regional GDP).

The errors with variance ***σ***_***y***_^**2**^ represent the natural “within-cluster variation” of petrochemical facilities, with level-2 structure errors reported by ***σ***_***α***_^**2**^.

The overall statistical validity of each model was confirmed by two Chi-Square Likelihood Ratio tests. The first compares the multilevel with a null (Intercept only) model, ensuring that any gains in predictive power sufficiently compensate for the added complexity. The second determines the significance of including hierarchical effects, by comparing the full model to a model constructed with only the fixed effects (Galwey, 2007, p.213–214).

Under the Bayesian framework, the performance of each parameter is assessed. The Gelman-Rubin potential scale reduction factor (PSRF) evaluated MCMC convergence, by comparing the variance between Markov chains to the variance within the chains for each modelled parameter (Gelman and Rubin, 1992). Adequate parameter convergence was observed in all models, with Gelman-Rubin PSRF values ≤1.05 (Gelman and Rubin, 1992). Checks were made for serial autocorrelation in the parameter simulation samples across 10 lag intervals, and this was shown to be minimal in all models (±0.01).

## 3 Results

### 3.1 The European petrochemical landscape

The current landscape of the European petrochemical industry remains in part reflective of its historical roots, which in the 1950's saw the strategic location of refineries around seaports receiving feedstock from overseas, later supplemented in the 1970's by North Sea petroleum reservoirs.

**Fig. 2:**
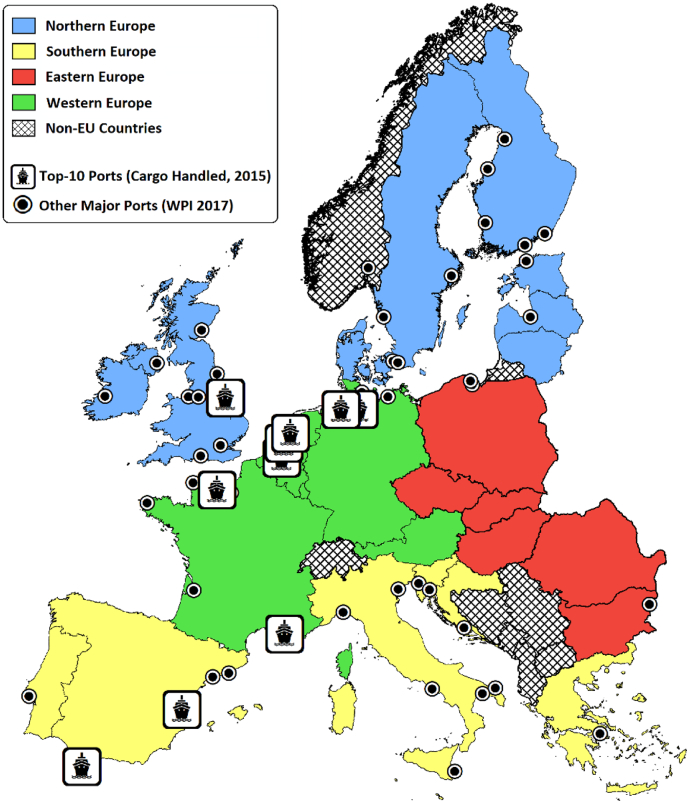
Major European Ports (Eurostat 2015, World Port Index 2017).

This legacy is observed by the presence of petrochemical clusters in six out of the ten most active European ports, where upper estimates indicate that the industry is accountable for 34–64% of handled cargo (Fig. 2, Appendix 2). These core locations include the historically important North Sea trade ports of Antwerp, Le Havre, Rotterdam and Immingham, and the Mediterranean ports of Algeciras and Marseille. Three ports contain chemical parks registered by the European Chemical Site Promotion Platform (ECSPP), which promotes integration and innovation across the industry.

**Fig. 3:**
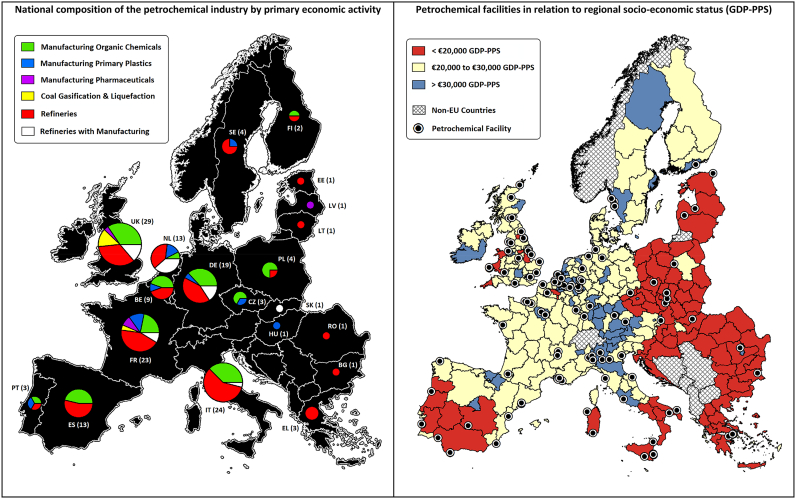
The European petrochemical landscape (facilities operating between 2007 and 2015).

Fig. 2, Fig. 3 illustrate this tendency for facilities to cluster either around these historically important trade ports, or close to existing coastal industrial infrastructure in less affluent regions. The petrochemical industry is also prominent within the Mediterranean islands of Malta and Sardinia, which are strategically positioned to process crude from Northern Africa and the Middle East for markets in mainland Europe. At present, 66% of European refineries are located within 10 km of major trade ports. Although coastal ports still dominate the petrochemical landscape, there has been a gradual shift towards inland markets, which Molle (1984) previously recorded to account for 15% of the market during 1950–60, and 28% in 1980. For instance, the port of Genoa has become an important terminal that feeds refineries within the Milan-Turin-Genoa industrial triangle of north-western Italy, via a complex network of pipelines.

Of the 156 facilities involved in upstream and downstream operations, 28% are in affluent regions (GDP-PPS > €30,000 per capita) and 27% are situated within relatively disadvantaged regions (GDP-PPS < €20,000 per capita). The most polluting facilities tend to be located within these financially disadvantaged regions, which report median benzene emissions of 12.6 t per annum, compared to facility emissions of 5.1 t per annum in affluent regions. 60% of the facilities in disadvantaged regions are near ports, compared to 47% of the facilities within affluent regions. In these affluent regions, 34% of the petrochemical industry focuses on manufacturing and 66% involve the refining of raw materials. In contrast, operations in disadvantaged regions are evenly split between manufacturing and refinery processes.

In terms of geographical differences, the Western and Northern European markets are more likely to involve the use of speciality chemicals, with 100% of the continent's petrochemical based pharmaceuticals and 73% of primary plastic products manufactured in these regions. There are several distinct differences in the composition of the sub-continental markets, with 54% of the facilities in Eastern Europe manufacturing basic organic chemicals, compared to only 39% of the facilities in Northern and Southern Europe. Refining appears most prolific in Northern and Southern Europe, where it accounts for up to 68%of the regions petrochemical activity. Refineries that use gasification and liquefaction techniques are almost exclusively found within the UK.

Table 2 provides a summary of petrochemical facility attributes, in accordance to facility size. 71% of the largest facilities (>1km^2^) are found near port locations, compared to only 45% of small facilities (<0.5km^2^). The largest operations typically operate out of regions that are financially deprived and experience the highest level of environmental burdens from the petrochemical industry. The presence of a larger facility within a region appears to promote the clustering of other petrochemical operations, perhaps attracted by the existing infrastructure and possibility of integration. The smallest facilities focus on manufacturing processes and tend to operate independently – only 29% are located within petrochemical clusters. In terms of finances, 7% of small and 4% of medium size facilities are at risk of meeting their long-term debts. While this remains a low risk, it is non-existent within larger petrochemical operations (> 1 km^2^ in size), highlighting the financial importance of scalability and supply chain integration through clustering.

**Table 2:** Summary of petrochemical operation attributes by facility footprint, where “Small” is <0.5km^2^, “Medium” = 0.5-1 km^2^, “Large” = 1–2 km^2^, and “Extra-Large” >2km^2^.

Characteristic	Description	Facility Extent				Total (N=156)
		Small (N=56)	Medium (N=45)	Large (N=45)	Extra-large (N=10)	
**Port Proximity**	**Near** (< 10 km)	45%	51%	73%	60%	56%

**Settlement Proximity**	**Urban** (< 0.5 km)	59%	44%	40%	60%	49%
	**Suburban** (0.5 to 5 km)	36%	49%	53%	30%	44%
	**Rural** (> 5 km)	5%	7%	7%	10%	6%

**Exposed Population**	**High:** > 7,500 residents within 1km	5%	11%	9%	0%	8%

**Economic Activity**	**Refineries**	25%	53%	73%	50%	49%
	**Manufacturing**	70%	38%	18%	0%	41%
	**Mixed**	5%	9%	9%	50%	10%

**NUTS2 Regions**	**GDP: High** (> €30,000)	30%	38%	18%	20%	43%
	**Petrochemical Cluster** (> 3 Facilities)	29%	29%	38%	40%	32%
	**Petrochemical Emissions: Low** (< 25 t/y Benzene)	63%	42%	18%	0%	39%

**European Sectors** **(UN Classification)**	**Western** (BE, DE, FR, NL)	41%	49%	38%	20%	41%
	**Eastern** (BG, CZ, HU, PL, RO, SK)	13%	4%	2%	10%	7%
	**Northern** (FI, LV, SE, UK)	25%	18%	22%	60%	24%
	**Southern** (EL, ES, IT, PT)	21%	29%	38%	10%	28%

**Parent Company**	**Independence: Low** (w/Majority Shareholder)	80%	78%	62%	80%	74%
	**Corporate Group: Large** (> 500 Companies)	27%	47%	49%	40%	40%
	**Solvency Ratio: Negative**	7%	4%	0%	0%	4%
	**Assets per Employee: Low** (< €2,500)	80%	78%	71%	60%	75%
	**Profit Margin: Negative**	21%	36%	40%	20%	31%

### 3.2 Polluting characteristics

This section examines the relationship between benzene emissions and the operational practices of 156 petrochemical facilities across Europe, with the intention of understanding how activity, location, and financial decisions may influence environmental outcomes.

An initial model was constructed to examine the influence of on-site activity (Model 0). As site-specific information on the volume of processed petroleum products is not publicly available, this was determined by the spatial extent of each facility. On-site activity was found to be accountable for 39% of the variation in benzene releases and was used as a controlling factor throughout the analysis. A further two models were used to inform the final model of noteworthy site-specific (Model 1) and corporate (Model 2) influences on benzene emission rates. Appendix 2 contains a detailed summary of the model performance metrics, with Appendix 3 providing the parameter coefficients of each model.

The final model of polluting characteristics within the petrochemical industry (Model 3), reveals that 67% of the benzene emissions released from European facilities can be predetermined by general characteristics of the industry. The remaining 33% of emissions releases appear to be a consequence of site-specific practices, which remain confidential or may even be unquantifiable. These include the presence of recycling, efficiency of emission abatement technology, frequency of emergency releases, and levels of employee due diligence.

**Fig. 4:**
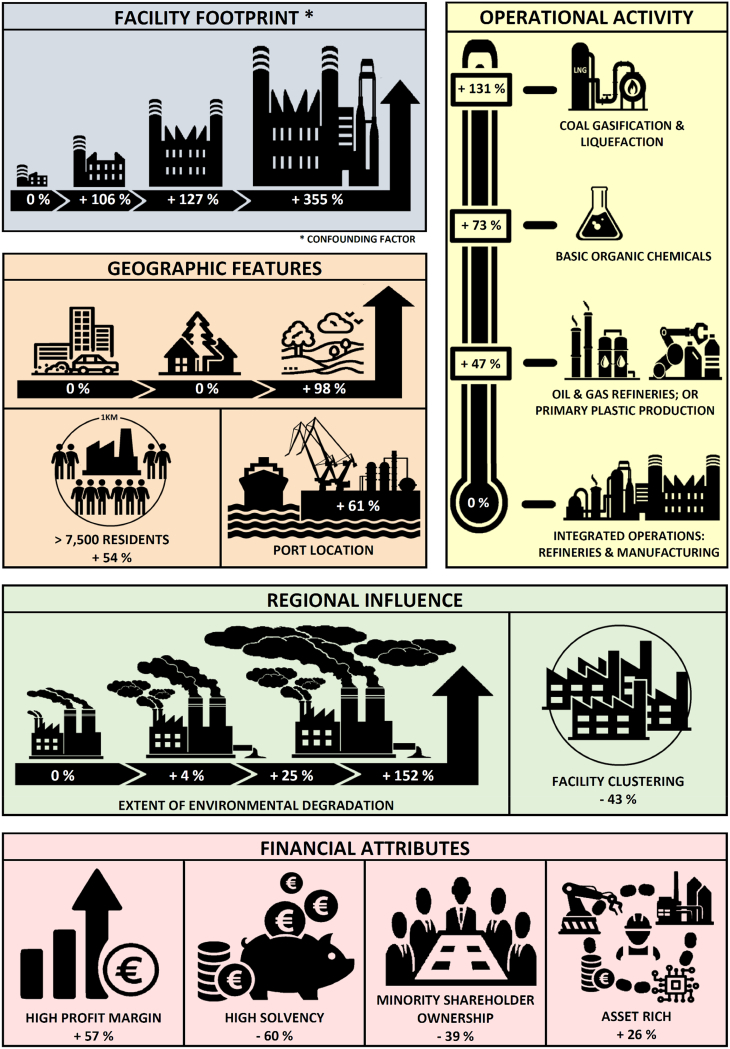
Iconographic interpretation, revealing how operational, geographic and financial measures influence the estimated baseline of emissions released from a European petrochemical facility (39.1 t per annum).

Fig. 4 provides an iconographic interpretation of the final model, revealing how modelled operational, geographic and financial measures influence the estimated baseline of emissions released from a European petrochemical facility (39.1 t per annum).

An incremental increase in emissions is observed in relation to facility activity, with extra-large facilities emitting 125.1 [94.2 to 157.0] tonnes per annum more than a small facility. The interaction effect between facility size and port location tends to identify coastal facilities as the highest polluters. This effect is inverse to size, with medium, large and extra-large port-based facilities respectively emitting an additional 24.0, 19.5 and 13.7 t per annum than their inland counterparts.

A rural-urban gradient is observed, with facilities positioned inside of, or within 5 km of an urban settlement emitting 38.2 [18.8 to 57.8] tonnes per annum less than their rural counterparts. While the most polluting practices tend to locate away from urban centres, a noticeable increase in emissions is recorded at a small number of facilities that have densely populated fence-line communities (> 7,500 residents within a 1 km catchment area). Interestingly, the 12 facilities in question are all located within major urban settlements, that are of moderate to high-affluence (€26,400 to €46,100 GDP-PPS).

In terms of production, the most polluting petrochemical operations are gasification and liquefaction refineries, followed by sites that manufacture basic organic chemicals, which respectively emit 32.6 [8.0 to 57.1] and 10.0 [0.1 to 19.8] tonnes per annum more than a traditional oil and gas refinery. The level of emissions released from the manufacture of primary plastics or petrochemical based pharmaceutical products was not found to significantly differ from that of a traditional refinery. Facilities that have combined the refining and manufacturing processes are shown to have a lower polluting potential than two independent operations, representing an overall saving of up to 28.5 [3.1 to 54.7] tonnes per annum. Future environmental gains should be achieved through greater integration of the refinery and manufacturing processes, if sterile preparation areas are not required.

Interestingly, the financial decisions of the parent company are shown to influence site-specific operations. Parent companies with a majority shareholder appear to have a reduced sense of environmental accountability, with their facilities emitting 15.1 [4.6 to 25.9] more tonnes per annum than operations with greater financial independence. Asset rich companies (> €2,500 per employee) are found to typically emit 10.1 [0.1 to 20.5] tonnes more per annum. The ability of a company to meet its long-term debt obligations also feeds into its individual operations, with facilities owned by solvent parent companies emitting 23.4 [0.5 to 46.1] fewer tonnes per annum than those that are insolvent. In contrast, companies with positive profit margins (i.e. short-term financial gains), ranging from 1 to 15% and 15–30%,operate facilities that respectively emit an additional 10.5 [0.1 to 21.0] or 22.2 [0.1 to 44.8] tonnes per annum. One may conclude, that parent companies with successful long-term business strategies tend to incorporate ‘greener’ operational practices.

An evaluation of regional influences found no association between polluting practices and affluence, represented by gross domestic product per capita in purchasing power standards (GDP-PPS). Facilities within a regional petrochemical cluster tended to emit 16.9 [6.8 to 26.8] fewer tonnes per annum, indicating that gains in environmental efficiency may be achieved from the integration of operations. On the other hand, emission rates were found to incrementally increase in relation to the regional extent of environmental degradation caused by the petrochemical industry. Operations located in regions where the overall environmental burden of the petrochemical industry is low, typically emit 59.4 [38.8 to 79.9]tonnes per annum less than facilities located in the most polluted regions, after controlling for facility size and density. This would imply that environmental accountability is influenced by the ‘good’ practice of neighbouring facilities.

### 3.3 Pollution events

The following analysis explores the relationship between maximum hourly benzene concentrations recorded by monitoring stations in each NUTS2 region, and total emissions from the petrochemical industry. The analysis considers, and controls for the impact of transport, which often occurs alongside industrial operations.

**Fig. 5:**
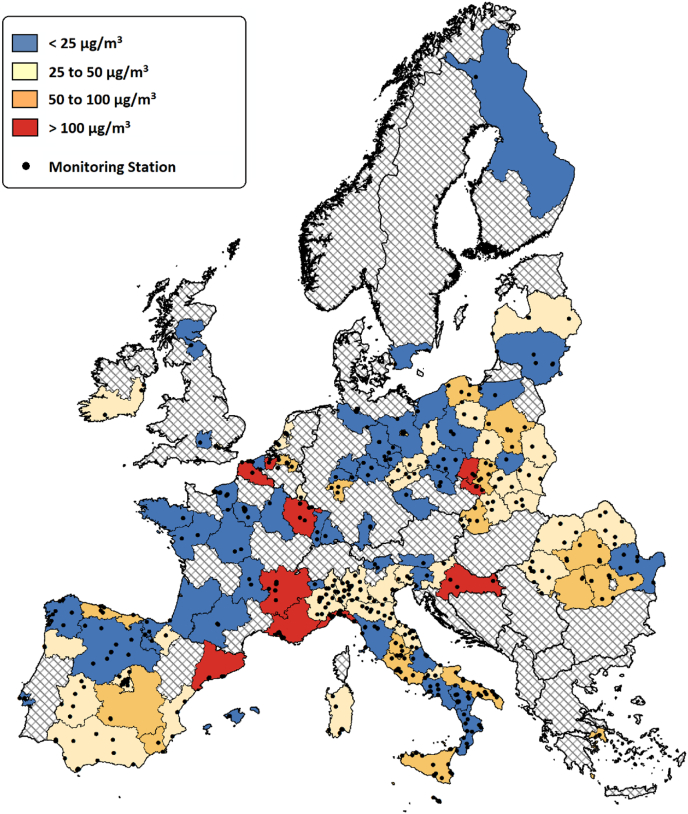
The maximum 1-hour benzene concentration of NUTS2 regions in 2013–2015, reported by monitoring stations with >50% annual capture rates.

Fig. 5 displays the maximum 1-hour benzene concentration of NUTS2 regions in 2013–2015, reported by monitoring stations with >50% annual capture rates. Across this period, several NUTS2 regions are identified to record a maximum 1-hour benzene concentration of >100 μg/m^3^. Provence-Alpes-Côte d'Azur, in southern France contains a cluster of 6 petrochemical operations around Martigues and Marseille, which collectively emit a high level of benzene (171 t/y). The region also experiences high levels of NMVOC emissions from HGVs (>500 t/y). Nord-Pas-de-Calais, in northern France contains a cluster of 3 petrochemical operations that jointly emit high levels of benzene (128 t/y) and experiences high levels of NMVOC emissions from HGVs. Substantial petrochemical activity in the eastern French regions of Rhône-Alpes (29 t/y) and Lorraine (31 t/y), are also found to coincide with 1-hour benzene concentrations >100 μg/m^3^. Of the two, only Rhône-Alpes experiences high contributions from a single form of road-transport - HGVs.

Benzene episodes in East Flanders (Belgium), Moravian-Silesian (Czech Republic) and Opolskie (Poland) are only partially explained by relatively low benzene contributions from the petrochemical industry (<25 t/y). still, all three regions are relatively unburdened by NMVOC emissions from various forms of road-transport (Buses <100 t/y, Diesel-cars <250 t/y, HGVs <500 t/y). In contrast, benzene episodes in Catalonia (Spain) are associated with relatively low contributions from the petrochemical industry (<25 t/y) and high levels of NMVOC emissions from HGVs. Finally, the regions of Liguria (Italy) and Continental Croatia contain no immediate or surrounding petrochemical activity, yet both exceed the hourly maximum of 100 μg/m^3^. This is likely attributed to the high levels of NMVOC emissions from HGVs. The coastal region of Liguria is also likely to be burdened by shipping emissions from the port of Genoa, which is an important tanker terminal.

Table 3 shows the pairwise correlation between pollution events and petrochemical emissions, or individual modes of road-transport. Emissions from petrochemical facilities are shown to have a moderate influence on benzene pollution events (P < 0.01). Only road-transport modes that run on diesel fuels were found to influence benzene pollution events, albeit in a minor way (P < 0.05).

**Table 3:** Maximum recorded 1-hour benzene concentration (2013–2015).

Test	Pairwise Correlation	DF	Spearman’s Rho	P-Value
**1**	(A) Maximum 1-hour Benzene (B) Petrochemical industry benzene emissions	116	0.38	< 0.01

**2**	(A) Maximum 1-hour Benzene (B) Road-transport: Bus NMVOC emissions	116	0.24	< 0.01

**3**	(A) Maximum 1-hour Benzene (B) Road-transport: HGV NMVOC emissions	116	0.16	0.04

**4**	(A) Maximum 1-hour Benzene (B) Road-transport: Petrol Car NMVOC emissions	116	0.05	0.31

**5**	(A) Maximum 1-hour Benzene (B) Road-transport: Diesel Car NMVOC emissions	116	0.17	0.03

A regression approach was then used to untangle these relationships, considering the interaction effect between industry and transport. Table 4 contains the significant outputs from the Bayesian Linear multilevel model, relating regional petrochemical and transport activities to the 1-hour maximum benzene concentration. The model includes the main sources of benzene emissions at a regional level (listed in Table 3), and the multilevel structure crudely considers for national meteorological differences. In total, 59% of the variation is accounted for, with the rest potentially explained by local pollutant sources (i.e. landfill and petrol pumps) and localised climatic conditions, which influence the dispersion of pollutants.

**Table 4:** Bayesian linear multilevel model relating petrochemical and transport activity to the 1-h maximum (r-squared = 0.59) and annual mean (r-squared = 0.30) benzene concentrations recorded across the NUTS2 regions (2013–2015).

Parameter Group	Categorical Contrast	Benzene concentration (μg/m^3^)	
		1-hour Maximum	Annual Mean
**0. Baseline (Intercept)**	--	+ 51.2 [25.3 to 77.7]	+ 1.4 [1.2 to 1.7]
**1. Petrochemical Industry**^**(**^⁎^**)**^	Low vs. Zero emissions	+ 114.4 [73.8 to 115.1]	+ 0.4 [ 0.2 to 0.6]
**1. Petrochemical Industry**^**(**^⁎^**)**^	High vs. Zero emissions	+ 123.5 [50.2 to 197.7]	+ 0.5 [ 0.1 to 0.5]
**2. Bus**^**(**^⁎⁎^**)**^	High vs. Low emissions	n/s	n/s
**3. Diesel Car**^**(**^⁎⁎^**)**^	High vs. Low emissions	n/s	n/s
**4. HGV**^**(**^⁎⁎⁎^**)**^	High vs. Low emissions	n/s	+ 0.4 [0.1 to 0.7]

**Interaction effects,** **where P < 0.10**	High (1,3,4) & Low (2) vs. Zero (1) & Low (2,3,4) emissions	+ 748.2 [623.6 to 877.3]	n/s
	High (1,2,3,4) vs. Zero (1) & Low (2,3,4) emissions	n/s	+ 1.4 [1.2 to 1.7]

**n/s** Insignificant coefficient at the 90% highest density interval, ⁎⁎⁎⁎ HGV NMVOCs: Low (≤ 500 t/y), High (> 500 t/y)

⁎ Petro-Industry Benzene: Zero (0 t/y), Low (≤ 25 t/y), High (> 25 t/y)

⁎⁎ Bus NMVOCs: Low (≤ 100 t/y), High (> 100 t/y)

⁎⁎⁎ Diesel-Car NMVOCs: Low (≤ 250 t/y), High (> 250 t/y)

The model confirms that petrochemical facilities are a key determinant of regional benzene pollution events, which are thought to be more detrimental to health than cumulative exposure (US EPA, 2012). The magnitude of these events incrementally increases with regional petrochemical release rates, although the most severe episodes appear when elevated petrochemical releases are combined with high levels of diesel traffic. Road-transport alone is not found to influence benzene pollution events.

Emissions from the petrochemical industry were found to have a weaker association with annual mean concentrations of benzene in 2013–2015, with model outputs explaining only 30% of the variation (see Table 4). While, the presence of the petrochemical industry was associated with a 29–36% increase in the annual mean concentration of benzene, no measurements (weighted and unweighted) from the 209 NUTS2 regions were in breach of European Directive 2008/50/EC, which established an annual average legal limit for benzene at 5 μg/m^3^. There may be a need for harder limits to be set, or the legislation to be adjusted to prevent the occurrence of pollution episodes – in reference to the hourly limits for the industrial pollutant sulphur dioxide.

### 3.4 Triple-jeopardy and regional disparity?

Table 5 summarises the performance of the Pan-European multilevel models, which explore the influence of petrochemical industry emissions and social inequality on regional health outcomes. A further two fixed parameters were used to control for rural-urban and sub-continental differences, with country specific differences explained by the model's hierarchical structure.

**Table 5:** Multilevel model performances.

		10-year age by gender standardised mortality rates (per 100,000)		Model B: Life expectancy 2006-15⁎⁎
		Model A1: All Causes	Model A2: Malignant Neoplasms (C00-97)⁎	
**Model Description**	Observations (N)	267	233	267
	Log Likelihood	-1,673	-1,012	-320

**Pseudo R-Squared** (**Nagelkerke, 1991**)	Full Model	0.75	0.69	0.90
	Partial Model	0.60	0.34	0.73

**Chi-Square Likelihood** **(P-Value)**	Full Model	< 0.01	< 0.01	< 0.01
	Hierarchical Effects	< 0.01	< 0.01	< 0.01

⁎ Malignant neoplasms diagnosed as “C00-C97” by the International Statistical Classification of Diseases and Related Health Problems, revision 10 (ICD-10)

⁎⁎ European Commission (EC) Eurostat estimates of average life expectancy for persons born between 2006 and 2015

The mortality and life expectancy models provide an excellent goodness-of-fit to the data, with the four fixed parameters explaining over 70% of the variation. The overall statistical validity and spatial component of each model is confirmed by Chi-Square Likelihood Ratio tests (P < 0.001). The hierarchical structure reveals that 15% of the variation in rates of regional mortality, from all causes, is a result of national differences – likely associated with lifestyle choice, access to and the provision of healthcare. National influences appear more prominent in mortalities attributed to malignant neoplasms, explaining 35% of the variation.

Financial and environmental trends were identified in relation to life expectancy and mortality rates at a regional level, after controlling for urban, national and sub-continental geographic structures (Table 6).

**Table 6:** Bayesian Linear multilevel model of NUTS2 regional health disparities.

Parameter Group	Categorical Contrast	10-year age by gender standardised mortality rates (per 100,000)		Model B: Life expectancy 2006-15
		A1: Mortality (All)	A2: Mortality (C00-C97)	
**Baseline (Intercept)**	--	+ 1,419 [1,302 to 1,534]	+ 296 [274 to 319]	+ 76.5 [75.2 to 77.9]

**GDP per capita in Purchasing** **Power Standards (GDP-PPS)**⁎	Very Deprived vs. Moderate levels of GDP	+ 96 [45 to 146]	n/s	- 1.2 [-1.5 to -0.8]
	Deprived vs. Moderate levels of GDP	+ 61 [21 to 103]	+ 8 [1 to 14]	- 0.8 [-1.1 to -0.6]
	Affluent vs. Moderate levels of GDP	n/s	n/s	+ 0.3 [0.1 to 0.6]
	Very Affluent vs. Moderate levels of GDP	-55 [-106 to -4]	n/s	+ 0.4 [0.1 to 0.7]

**Petrochemical Industry:** **Benzene Emissions (t/yr.)**	< 25 tonnes vs. 0 tonnes	+ 44 [8 to 82]	n/s	n/s
	25 - 225 tonnes vs. 0 tonnes	+ 57 [11 to 106]	+ 7 [0 to 14]	- 0.3 [-0.6 to -0.1]
	> 225 tonnes vs. 0 tonnes	+ 185 [82 to 293]	+ 35 [12 to 58]	- 1.4 [-2.0 to -0.7]

**Urban-Rural Divide**	High vs. Low Population Density	n/s	+ 9 [3 to 15]	- 0.3 [-0.5 to 0.0]

**European Sub-Continent** **(Geographic Disparities)**	Northern vs. Eastern	- 306 [-455 to -155]	- 46 [-79 to -12]	+ 2.6 [0.9 to 4.4]
	Southern vs. Eastern	- 433 [-587 to -289]	- 64 [-98 to -32]	+ 4.9 [3.2 to 6.8]
	Western vs. Eastern	- 425 [-580 to -269]	- 37 [-69 to -6]	+ 4.6 [2.8 to 6.5]

**n/s:** Insignificant coefficient at the 90% highest density interval.

⁎ **GDP-PPS**: Very Deprived (< €20,000); Deprived (€20,000 to €25,000); Moderate levels of GDP (€25,000 to €30,000); Affluent (€30,000 to €35,000); Very Affluent (> €35,000)

An incremental increase in total mortality rates is observed in response to decreasing financial prosperity, with very-deprived (< €20,000 GDP-PPS) and deprived (€20,000 to €25,000 GDP-PPS) regions respectively reporting an additional 96.3 and 61.3mortalities per 100,000 persons. It is estimated that these same regions experience a 1.2 and 0.8 reduction in life expectancy per person, respectively. In contrast, persons from affluent (€30,000 to €35,000 GDP-PPS) and very-affluent (> €35,000 GDP-PPS) regions are estimated to respectively experience life expectancy gains of 0.3 and 0.4 years.

Higher levels of benzene emissions from petrochemical operations were associated with an increase in overall mortality rates – with an additional 44.2, 57.0 and 185.3 mortalities per 100,000 persons, respectively reported in regions emitting 1–25 tonnes, 25 to 225 tonnes and >225 tonnes per annum.. Meanwhile, a reduction in life expectancy and mortalities attributed to malignant neoplasms is only associated with petrochemical activity, in regions where the industry releases >25 t/y. Life expectancy is estimated to reduce by 0.3 and 1.4 years in regions where the petrochemical industry respectively emits 25 to 225 tonnes and >225 tonnes per annum.

**Fig. 6:**
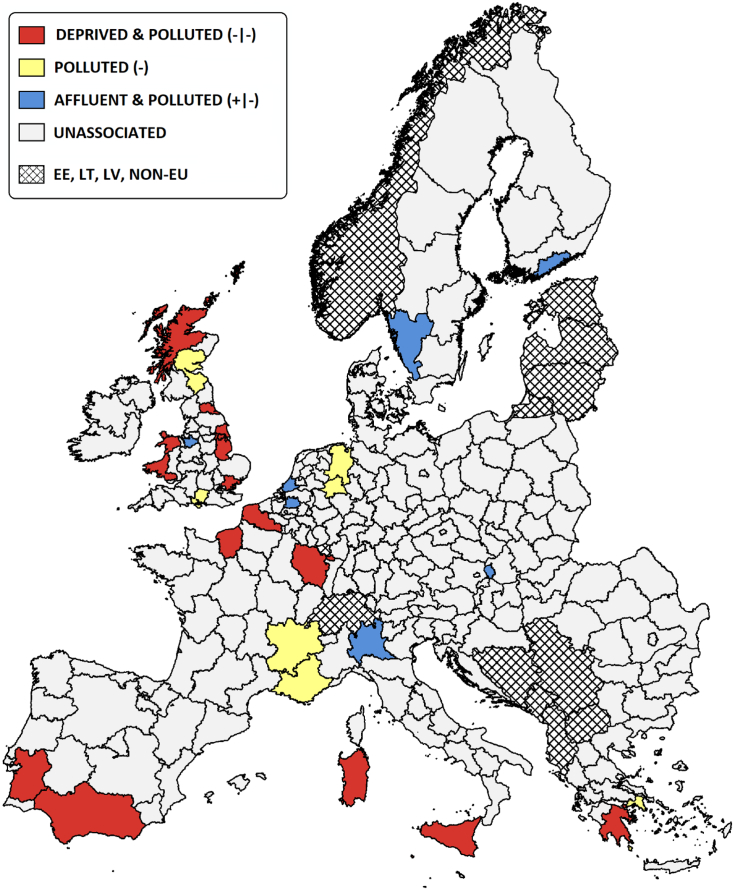
Regional differences in life-expectancy, attributed to socio-economic and environmental influences.

Fig. 6 provides an illustrative cardinality count of the gains and losses in life-expectancy from regional socio-economic and environmental influences. An expected decrease in life-expectancy from the coexistence of unfavourable financial and environmental influences from the petrochemical industry (coloured red), is observed in 14 regions. In contrast, there are only 7 regions where the residents are identified as relatively affluent but environmentally burdened by the petrochemical industry (coloured blue). Although a higher proportion of deprived communities are thought to be impacted by the petrochemical industry, a “triple jeopardy” of social, environmental and health inequalities is not universally reported. Historically, the petrochemical industry has also tended to locate at ports along the Mediterranean and North Sea in relatively affluent countries. The existence of a few, polluted but affluent petrochemical regions, confirms that this prevailing structural legacy exists beyond the “triple jeopardy” threat.

## 4 Discussion

Our Pan-European analysis offers a framework for identifying, evaluating, and comparing local epidemiological studies of benzene exposures in the European petrochemical industry. Bayesian multilevel modelling reveals patterns of regional inequality in polluting practices and environmental exposures across Europe, despite the presence of apparently robust and harmonized regulations. These findings resonate with patterns of environmental justice research around the world, where the heaviest burdens of toxic exposure are concentrated in the poor, ethnic minority, and disadvantaged communities (Bullard, 1994; Morello-Frosch et al., 2002; Walker, 2012).

156 petrochemical facilities were identified from the European Pollutant Release and Transfer Register (E-PRTR), 51% of which are involved in the ‘upstream’ refinery process of fossil fuels. An examination of polluting practices revealed, that 67% of the benzene emissions at any given facility are predetermined by characteristics found throughout the petrochemical industry. The remaining 33% of emissions released appear to be a consequence of site-specific practices, which remain confidential or may even be unquantifiable (i.e. recycling, abatement technology and due diligence).

Facilities located close to ports were found to emit up to 61% [39–174%] more carcinogenic pollutants than their counterparts at inland locations. The discovery of an urban-rural gradient shows that the industry has some social accountability, with the most polluting practices tending to locate away from major settlements. However, a 54% [9–98%] increase in emissions was recorded at a small number of urban facilities with densely populated fence-line communities (> 7,500 residents within a 1 km catchment area).

Furthermore, the financial decisions of the parent company are shown to influence site-specific operations. Parent companies owned by a majority shareholder appear to have a reduced level of environmental accountability, with their facilities emitting 39% [12–66%] more than operations which are financially independent from their shareholders. Parent companies with successful long-term business strategies tended to incorporate ‘greener’ operational practices. By contrast, businesses looking for short-term gains emitted between 27 and 57% more carcinogens. Industrial pollution episodes have previously been documented prior to the closure or mothballing of plants (Bhopal et al., 1994), perhaps as a last-minute attempt to meet the demands of creditors or a decline in maintenance.

Clusters of petrochemical operations were found to emit 43% [17–68%] fewer carcinogens, demonstrating the potential gains in environmental efficiency through the integration of operations. On the other hand, facility emissions were found to increase as regional level of environmental degradation from the petrochemical industry rose. The extent of environmental accountability may decline by 152% [99–204%] if ‘good’ practice is not adhered to by neighbouring facilities.

On paper, facilities in relatively disadvantaged regions (< €20,000 per capita) have a higher overall polluting potential, typically emitting 12.6 tonnes of benzene per annum, which is substantially higher than the 5.1 tonnes emitted by facilities located in affluent regions (> €30,000 per capita). However, upon disentangling the various operational characteristics, no significant differences were found in relation to regional socioeconomic status. These differences appear to be indirectly captured by other measures: the manufacture of organic chemicals is more prevalent in deprived areas (40% vs 29% of facilities in affluent locations), which are often in port locations (60% vs 34%) and tend to be burdened by the runaway effect of environmental degradation from the industry at a regional level (14% vs 0%). In addition, there are no refineries from affluent regions that use gasification and liquefaction, which were identified as the most polluting activities within the petrochemical industry.

The analysis then explored the connection between benzene pollution events and regional emissions from the petrochemical industry, controlling for national meteorological differences and the impact of transport, which often occurs alongside industrial operations. In total, 59% of the variation was accounted for, with the rest potentially explained by localised pollutant sources (i.e. landfill and petrol pumps) and climatic conditions. Petrochemical facilities were confirmed to be a key determinant of regional benzene pollution events, which are thought to be more detrimental to health than cumulative exposures to low concentrations (US EPA, 2012). The magnitude of these events incrementally increases with regional petrochemical release rates, although the most severe episodes appear when elevated petrochemical releases are combined with high levels of diesel traffic. Road-transport alone was not found to influence benzene pollution events.

To conclude, attention was shifted towards the petrochemical industry and the potential presence of social, environmental and health inequalities. The mortality and life expectancy models provide an excellent goodness-of-fit to the data, explaining >70% of the variation. Financial and environmental trends were associated with a decrease in life-expectancy and regional mortality rates, after controlling for urban, national and sub-continental geographic structures. However, a causal link cannot be established from an investigation of small-area population units, which may be influenced by ecological fallacy – that is, an association between variables on the aggregate level does not necessarily represent an association at the individual level. This bias occurs because vital statistics do not characterise, within and between areas, the variability in exposure to environmental contaminants and potential confounders. While the analysis has solely explored the contributions from petrochemical facilities, consideration should also be made to the contributions of other industry that tend to cohabit these industrial zones, emitting a concoction of other pollutants. While it is beyond the scope of this Pan-European analysis, which examines trends in the petrochemical industry, perhaps it is advisable for localised case-studies to take a multipollutant approach. Nevertheless, the analysis has identified several interesting regional trends, providing a strong platform for the design of local confirmatory case-studies that target these areas of concern.

Table 7 summarises the existing investigative research that has been conducted within regions where petrochemical activity and socioeconomic factors are thought to collectively decrease life-expectancy. To date, research on residential exposures to the petrochemical industry has taken place in 12 out of the 14 “triple jeopardy” NUTS2 regions. The research has largely been conducted via an ecological framework (56%), with few studies drawing conclusions from large cohorts measured over several years (13%). Tracer pollutants, in the form of benzene or benzo[*a*]pyrene, have been used to quantify exposure to the petrochemical industry in only 12% of the research, with 50% using facility proximity. The remaining 38% of case studies use indirect environmental exposures, assessing the broader impact of local industry. Of the research measuring direct exposures, 50% focused on refineries, 10% on the downstream operations, and 40% across both sectors.

**Table 7:**
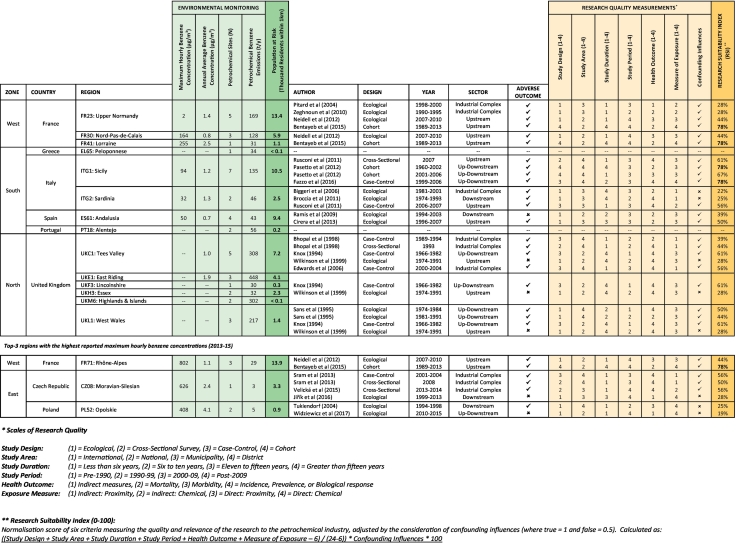
Investigative summary of the existing research on residential exposures to the petrochemical industry, in regions thought to be burdened by socioeconomic conditions and petrochemical activity (see Fig. 5) (Biggeri et al., 2006; Broccia et al., 2011; Cirera et al., 2013; Jiřík et al., 2016; Neidell and Lavaine, 2012; Ramis et al., 2009; Sram et al., 2013; Tukiendorf, 2004; Velická et al., 2015; Widziewicz et al., 2017; Zeghnoun et al., 2010).

In terms of recorded outcomes, 53% of the case studies asses the long-term effect of exposure to petrochemicals (i.e. mortality and prevalence rates), with 37% examining the near-term effects (i.e. hospitalisation and incidence rates). Other approaches include, Rusconi et al.'s (2011) use of biological responses to quantify the immediate response to refinery emissions in Sardina, and Pitard et al.'s (2004) investigation of preventative actions, which linked pharmaceutical sales to industrial pollution episodes in Rouen and Le Havre. Within the 14 priority regions, 88% of the cited literature identifies a connection between residential exposure to the petrochemical industry and directly observed health outcomes - hematologic malignancies (25%) and lung cancers (25%) are often diagnosed. However, 38% of the research was conducted prior to 1999 and is now quite dated in relation to current environmental regulation.

It should be noted that the 8 priority regions (57%) with functional air quality monitoring equipment (in addition to 3 regions that record the highest maximum hourly benzene concentrations) are compliant with European Directive 2008/50/EC, which established an annual average legal limit for benzene at 5 μg/m^3^. The highest reported annual concentration for 2013–15 is 3.3 μg/m^3^ in the Polish region of Opolskie. Perhaps it is time for harder limits to be set, or the legislation to be adjusted to prevent the occurrence of pollution episodes, which we have linked to petrochemical activity. For instance, other industrial pollutants such as sulphur dioxide already have an hourly limit set at 350 μg/m^3^, which cannot be exceeded >24 times a year.

The published evidence base was evaluated using a Research Suitability Index, which identified only 3 out of the 14 priority areas to contain epidemiological research on the petrochemical industry deemed to be of a high standard (RSS >70%). This knowledge is limited to the French regions of Upper Normandy, Lorraine, and Provence-Alpes-Côte d'Azur. In contrast, hardly any epidemiological research on the petrochemical industry has been conducted in the UK, since the national studies by Knox (1994) and Wilkinson et al. (1999).It is only Teesside (Bhopal et al., 1998; Edwards et al., 2006) and Wales (Sans et al., 1995) that have local case studies, the latter of which reported an 8% excess incidence of all cancers for residents <7.5 km from the now closed Baglan Bay petrochemical works. There is a need for new case-studies, and a national revaluation of the UK petrochemical industry is long overdue. It is also concerning that no case studies are present for 2 of the priority regions identified by our analysis, which includes operations close to Sines (Portugal) and Corinth (Greece), both of which have small fence-line communities.

Although the analysis has focused on emission of benzene from petrochemical facilities (because it is tracer pollutant of petrochemical activity, a known carcinogen, and has an existing albeit unreliable at times monitoring network), one should remember that several other carcinogenic and important European directive pollutants are emitted by this industry. Benzene emission rates are shown to be highly correlated with other Non-Methane Volatile Organic Compounds (NMVOCs), which are often harmful to health, but we are unable to reveal which additional components are of greatest importance as they are rarely measured on an individual basis (Table 8). Benzene is shown to account for up to 19% of the weight in tonnes, of NMVOCs emitted by European petrochemical facilities. A low level of correlation is also observed between benzene, sulphur dioxide (0.29) and carbon dioxide (0.35). While the release of NMVOCs European industry has decreased by approximately 37% on 1990 levels (EEA, 2014), these reductions have occurred as industry adapts to broader environmental regulation, which in recent years has tended to focus on climate issues. Our findings would indicate that there is still a need for tighter regulation on the release of carcinogens, to ensure that all communities have access to a healthy and sustainable environment.

**Table 8:** Spearman's pairwise correlation between benzene and other pollutants emitted by European petrochemical facilities (N = 156).

Pollutant	Facilities Monitored	Spearman’s Rho	P-Value
Non-Methane Volatile Organic Compounds (NMVOCs)	128 (82%)	0.65	< 0.01
• NMVOCs, excluding Benzene	128 (82%)	0.64	< 0.01
• Polycyclic Aromatic Hydrocarbons (PAHs)	10 (6%)	--	> 0.1
• Toluene	2 (1%)	--	--
• Xylenes	1 (1%)	--	--
• Ethylbenzene	0 (0%)	--	--
Nitrogen Oxides (NO_X_)	127 (81%)	0.24	< 0.01
Carbon Dioxide (CO_2_)	108 (69%)	0.35	< 0.01
Sulphur Dioxide (SO_2_)	98 (63%)	0.29	< 0.01
Particulate Matter (PM_10_)	57 (37%)	0.23	0.04

## 5 Conclusion

This article presents the first Pan-European analysis of the petrochemical industry, connecting petrochemical activity to disparities in regional mortality rates, and identifying measures of best environmental practice that may be implemented to mitigate such adverse outcomes.

Our findings have several policy implications. Firstly, our estimates suggest that benzene pollution episodes are linked to increased activity within the petrochemical industry. While all regions are compliant with the annual average benzene limit of 5 μg/m^3^, the strengthening of regulation towards an hourly or daily limit is advocated. Secondly, our ecological analysis implies that people located near refineries or petrochemical complexes experience a higher rate of adverse health effects leading to mortality, with disproportionate environmental hazards often found where the poorest populations reside. These regional trends were largely confirmed by the existing epidemiological literature, but we also identified under-researched regions that require further investigation. The research demonstrates that problems of environmental injustice extend to the European context, despite European-wide environmental and health regulations. Finally, our analysis of uneven polluting practices highlights different industrial and regulatory strategies for reducing pollution. We recommend strengthening the regulation of benzene in addition to other toxic petrochemicals (volatile organic compounds, among others) to mitigate persistent regional inequalities in environmental exposures.

## Figures and Tables

**Table:** 

Age group	European standard Population: 2015		Annual deaths (all causes)		Population		Standardised mortality rates		
	Male	Female	Male	Female	Male	Female	Male	Female	Total
<5	2,637	2,503	75	58	79,266	75,066	2.5	1.9	4.4
5–9	2,724	2,587	6	4	70,714	67,339	0.2	0.2	0.4
10–14	2,645	2,512	8	5	66,832	63,735	0.3	0.2	0.5
15–19	2,731	2,587	22	10	74,672	72,145	0.8	0.4	1.2
20–24	2,988	2,865	52	23	106,258	112,192	1.5	0.6	2.1
25–29	3,193	3,121	76	32	134,322	137,724	1.8	0.7	2.5
30–34	3,330	3,294	91	44	130,420	125,677	2.3	1.2	3.5
35–39	3,449	3,412	133	69	126,743	118,351	3.6	2.0	5.6
40–44	3,581	3,559	260	130	142,006	132,728	6.6	3.5	10.1
45–49	3,705	3,708	472	230	142,598	135,136	12.3	6.3	18.6
50–54	3,570	3,630	691	359	121,295	118,797	20.3	11.0	31.3
55–59	3,270	3,416	932	491	103,425	108,054	29.5	15.5	45.0
60–64	2,932	3,173	1,251	705	93,089	99,951	39.4	22.4	61.8
65–69	2,569	2,874	1,843	1,082	93,509	103,095	50.6	30.2	80.8
70–74	1,999	2,383	2,422	1,639	81,332	97,656	59.5	40.0	99.5
75–79	1,627	2,163	2,358	2,082	50,227	69,611	76.4	64.7	141.1
80–84	1,085	1,686	2,039	2,720	25,677	48,487	86.2	94.6	180.7
85+	779	1,713	2,430	7,289	16,052	51,926	117.9	240.4	358.4
Total	48,814	51,186	15,162	16,973	1,658,437	1,737,670	512	536	1,047

**Table:** 

Port	Geography		Cargo handled in 2015					Petrochemical facilities		Chemical park		
			Total		Liquid bulk goods							
	Country	European sector	Weight (Kt)	Rank	Weight (Kt)	Rank	Total proportion	Nearest	Cluster	Description	ECSPP member	Source
Rotterdam	NL	West	436,942	1	216,571	1	50%	< 5 km	Y	Rotterdam has been one of the world's major centres for oil and chemicals for several decades. Its strategic location and unrivalled pipeline access to markets all over Europe have led to the establishment of a modern and diverse cluster of oil and chemical activities.	Y	https://chemicalparks.eu/parks/port-of-rotterdam
Antwerpen	BE	West	190,107	2	66,123	2	35%	< 5 km	Y	The Port of Antwerp is home to the largest petrochemical cluster in Europe. Seven of the world's top ten petrochemical companies have one or more production units in the Antwerp Chemical Cluster. The Port of Antwerp can be defined as a diversified cluster handling containers and other general cargo.	Y	https://chemicalparks.eu/parks/port-of-antwerp
Hamburg	DE	West	120,172	3	14,020	16	12%	10 km	N	–	–	–
Amsterdam	NL	West	98,776	4	43,861	4	44%	> 50 km	N	The port area has a large cluster of specialty chemical companies, but their primary activity appears unrelated to the petrochemical industry.	Y	https://chemicalparks.eu/parks/port-of-amsterdam
Algeciras	ES	South	79,374	5	27,344	8	34%	< 5 km	Y	The port of Acerinox is primarily involved in the trade of commodities related to the production of stainless steel. The petrochemical complex in Guadarranque has capitalised on the established industrial infrastructure within the bay.	N	http://www.industrialalgecirasbay.com
Marseille	FR	West	77,479	6	49,933	3	64%	< 5 km	Y	The chemical industry in Provence includes a very wide range of activities from petrochemicals to specialty chemicals for the semiconductor industry. This activity was developed west of the city of Marseilles, from the industrial-port zone of Fos-Lavéra to the banks of the Etang de Berre lake, constituting one of the most important chemical centres in Europe.	N	https://chemicalparks.eu/organizations/provence-promotion
Le Havre	FR	West	62,947	7	40,070	5	64%	< 5 km	Y	A major refining and chemical complex positioned strategically in the petrochemical supply chain, with direct access to multimodal connexions (sea, rail, barge, pipe, road) and easy access to utility networks.	Y	https://chemicalparks.eu/parks/port-of-le-havre
Immingham	UK	North	59,103	8	21,301	12	36%	< 5 km	Y	The Humber is one of the UK's largest chemicals producing regions. The industry flourished around the estuary where it is easy to get raw materials in and finished products out. The Saltend Chemicals Park is located on the northern bank near Hull, and a cluster of refineries exist along the south bank between Immingham and Grimsby.	N	https://investhumber.com/invest/growth-sectors/chemicals
Valencia	ES	South	57,557	9	3,814	24	< 10%	> 50 km	N	–	–	–
Bremerhaven	DE	West	49,753	10	330	26	< 10%	35 km	N	–	–	–

**Table:** 

		Model 0: adjust activity	Model 1: facility attributes	Model 2: parent company	Model 3: final selection
Model description	Observations (N)	156	156	156	156
	Log Likelihood	−796	−752	−786	−749
Pseudo R-squared (Nagelkerke, 1991)	Full Model	0.39	0.65	0.47	0.67
	Unstructured model	–	0.59	0.45	0.59
Chi-square likelihood: P-value	Full Model	< 0.01	< 0.01	< 0.01	< 0.01
	Hierarchical Effects	–	< 0.01	0.05	< 0.01

**Table:** 

Parameter Group		Categorical Contrast	Model 0: Activity Adjustment	Model 1: Facility Attributes	Model 2: Parent Company	Model 3: Final Selection
**Baseline (Intercept)**		--	n/s	59.4 [30.9 to 88.0]	n/s	39.1 [13.6 to 64.4]
**Facility**	**Facility Size**	Medium vs. Small	18.4 [5.3 to 31.3]		24.6 [11.0 to 37.1]	
		Large vs. Small	25.4 [12.1 to 38.2]		37.6 [24.0 to 51.5]	
		Extra-Large vs. Small	138.9 [116.3 to 161.6]		140.0 [118.0 to 161.8]	
	**Facility Size** **x** **Port Proximity**	Medium Port vs. Small Port Facility		38.1 [21.0 to 54.1]		41.4 [25.9 to 58.0]
		Large Port vs. Small Port Facility		46.4 [30.1 to 62.7]		49.7 [33.4 to 65.8]
		Extra-Large Port vs. Small Port Facility		130.9 [103.4 to 160.1]		138.8 [110.4 to 166.2]
		Small Inland vs. Small Port Facility		n/s		n/s
		Medium Inland vs. Small Port Facility		15.5 [0.0 to 31.1]		17.4 [2.2 to 32.8]
		Large Inland vs. Small Port Facility		24.6 [5.0 to 43.9]		30.2 [11.2 to 49.4]
		Extra-Large Inland vs. Small Port Facility		126.3 [93.7 to 159.3]		125.1 [94.2 to 157.0]
	**Urbanisation**	Peri-Urban vs. Urban		n/s		n/s
		Rural vs. Urban		40.9 [21.4 to 60.9]		38.2 [18.8 to 57.8]
	**Exposed Population**	High vs. Low		15.2 [0.1 to 30.0]		21.1 [3.7 to 38.3]
	**Economic Activity**⁎	Coal Gasification & Liquefaction		44.6 [17.4 to 69.8]		32.6 [8.0 to 57.1]
		Refineries with Manufacturing		-16.9 [-33.2 to -0.6]		-18.6 [-34.9 to -3.0]
		Manufacturing Basic Organic Chemicals		n/s		10.0 [0.1 to 19.8]
		Manufacturing Pharmaceuticals		n/s		n/s
		Manufacturing Primary Plastics		n/s		n/s
**Regional**	**GDP-PPS** **(NUTS2)**	Low vs. High		n/s		
		Moderate vs. High		n/s		
	**Facility Density** **(NUTS2)**	High vs. Low		-17.5 [-30.2 to -4.8]		-16.9 [-26.8 to -6.8]
	**Facility Emissions** **(NUTS2)**	Low vs. Very-High		-70.7 [-96.6 to -45.9]		-59.4 [-79.9 to -38.8]
		Moderate vs. Very-High		-68.6 [-92.0 to -44.2]		-58.0 [-78.0 to -39.7]
		High vs. Very-High		-61.7 [-82.5 to -41.2]		-49.7 [-67.6 to -30.6]
	**European Sectors** **(UN Classification)**	Eastern vs. Western		n/s		
		Northern vs. Western		n/s		
		Southern vs. Western		n/s		
**Financial** **(2011-15)**	**BvD Independence**	High vs. Low			-19.5 [-31.2 to -6.9]	-15.1 [-25.9 to -4.6]
	**Corporate Group**	Large vs. Small			-11.3 [-22.7 to -0.4]	n/s
	**Solvency Ratio**	Negative vs. Positive			52.1 [24.4 to 80.1]	23.4 [0.5 to 46.1]
	**Assets ÷ Employees**	High vs. Low			13.0 [1.0 to 24.9]	10.1 [0.1 to 20.5]
	**Profit Margin**	Low vs. Negative			13.6 [1.3 to 26.2]	10.5 [0.1 to 21.0]
		High vs. Negative			24.1 [0.1 to 48.4]	22.2 [0.1 to 44.8]

Facility Size: Small (< 0.5km), Medium (0.5 to 1km), Large (1 to 2km), Extra-Large (> 2km)

Port Proximity: Port Facility (< 10km), Inland Facility (≥ 10km)

Urban Proximity: Urban (< 0.5km), Peri-urban (0.5 to 5km), Rural (> 5km from a settlement of > 5,000 people)

Exposed Population: Low (< 7,500 residents within 1km), High (≥ 7,500 residents within 1km)

NUTS2 - GDP-PPS: Low (< €20,000), Moderate (€20,000 to €30,000), High (> €30,000)

NUTS2 - Facility Density: Low (< 4 facilities), High (≥ 4 facilities)

NUTS2 - Facility Emissions: Low (< 25 t/y), Moderate (25 to 75 t/y), High (75 to 225 t/y), Very-High (> 225 t/y)

BvD Independence: Low (> 50% Single Shareholder Ownership), High (≤ 50% Single Shareholder Ownership)

Corporate Group: Small (< 500 companies), Large (≥ 500 companies)

Solvency Ratio: Negative (< 0%), Positive (≥ 0%)

Assets per Employee: Low (< €250,000), High (≥ €250,000)

Profit Margin: Negative (< 0%), Low (0% to 15%), High (> 15%)

⁎ Economic Activity Benchmark (vs. Refineries); n/s = Insignificant coefficient at the 90% highest density interval;

## Appendix 1 Calculation of standardised mortality rates

Standardised mortality rates were calculated using the demographic records of 9,936 gender-by-age-by-region subpopulations, extracted from the Eurostat census (https://ec.europa.eu/eurostat/data).

This procedure removes the confounding influence of gender and age-related risks, then standardises the adjusted-rates in accordance to an expected population structure.

To account for temporal fluctuations, a series of annual average death and population counts were calculated for the 10-year period of 2006–15.

For standardisation purposes, a 100,000 person ‘European Standard Population’ was constructed from the population of all EU-28 countries in 2015. This hypothetical division of the population into age-by-gender bands allows for the direct comparison of geographic areas.

The following example is provided for the NUTS-2 region of Berlin (DE30), which records an annual age-by-gender standardised mortality rate of 1,047 deaths per 100,000 persons from all causes:

## References

[bb0005] Bentayeb M, Wagner V, Stempfelet M, Zins M, Goldberg M, Pascal M, Larrieu S, Beaudeau P, Cassadou S, Eilstein D, Filleul L, Le Tertre A, Medina S, Pascal L, Prouvost H, Quénel P, Zeghnoun A & Lefranc A (2015). Association Between Long-Term Exposure To Air Pollution And Mortality In France: A 25-Year Follow-Up Study. Environ. Int., 85, Pp.5–14. 10.1016/j.envint.2015.08.006 26298834

[bb0010] Bhopal R., Phillimore P., Moffatt S., Foy C. (1994). Is living near a coking works harmful to health? A study of industrial air pollution. J. Epidemiol. Community Health.

[bb0015] Bhopal R., Moffatt S., Pless-Mulloli T., Phillimore P., Foy C., Dunn C., Tate J. (1998). Does living near a constellation of petrochemical, steel, and other industries impair health?. Occup. Environ. Med..

[bb0020] Biggeri A., Lagazio C., Catelan D., Pirastu R., Casson F., Terracini B. (2006). Environment and health in Sardinia, Italy. Epidemiology.

[bb0025] Broccia G., Longinotti M., Giannico B., Porcu C., Chessa E. (2011). Haematological malignancies on the island of Sardinia, 1974-1993: a geographical study. The Open Hematology Journal.

[bb0030] Bullard R. (1994). Unequal Protection: Environmental Justice and Communities of Color.

[bb0035] Cirera L., Cirarda F., Palència L., Estarlich M., Montes-Martínez A., Lorenzo P., Daponte-Codina A., López-Abente G. (2013). Mortality due to haematological cancer in cities close to petroleum refineries in Spain. Environ. Sci. Pollut. Res. Int..

[bb0040] Denwood M. (2016). Runjags: an R package providing Interface utilities, model templates, parallel computing methods and additional distributions for MCMC models in JAGS. J. Stat. Softw..

[bb0045] Department for Environment, Food and Rural Affairs (DEFRA) (2018). National Statistics Release: Emissions of Air Pollutants in the UK, 1970 to 2016.

[bb0050] Edwards R., Pless-Mulloli T., Howel D., Chadwick T., Bhopal R., Harrison R., Gribbin H. (2006). Does living near heavy industry cause lung cancer in women? A case-control study using life grid interviews. Thorax.

[bb0055] European Commission (2003). Regulation Number 1059/2003 - The establishment of a common classification of territorial units for statistics (NUTS). Available via: https://eur-lex.europa.eu/eli/reg/2003/1059/oj (Accessed: Monday 30th July 2018).

[bb0060] European Commission (2006). Regulation Number 166/2006 - The establishment of a European Pollutant Release and Transfer Register. Available via: http://data.europa.eu/eli/reg/2006/166/2009-08-07 (Accessed: Monday 30^th^ July 2018).

[bb0065] European Commission (2007). Directive 2007/2/EC - Establishing an Infrastructure for Spatial Information in the European Community (INSPIRE). Available via: https://eur-lex.europa.eu/eli/dir/2007/2/oj (Accessed: Monday 30^th^ July 2018).

[bb0070] European Environment Agency (EEA) (2014). Non-methane volatile organic compounds (NMVOC) emissions. EEA.

[bb0075] European Environment Agency (EEA) (2017). Air quality in Europe. EEA Report Number 13/2017.

[bb0080] European Environment Agency (EEA) (2018). *AirBase - The European air quality database*. Copenhagen, Denmark. Accessed: Monday 2^nd^ April.

[bb0085] Fazzo L, Carere M, Tisano F, Bruno C, Cernigliaro A, Cicero M, Comba P, Contrino M, DE Santis M, Falleni F, Ingallinella V, Madeddu A, Marcello I, Regalbuto C, Sciacca G, Soggiu M & Zona A (2016). Cancer incidence in Priolo, Sicily: a spatial approach for estimation of industrial air pollution impact. Geospat. Health, 11(320), pp.43–55. 10.4081/gh.2016.320 27087035

[bb0090] Galwey N. (2007). Introduction to Mixed Modelling: Beyond Regression and Analysis of Variance.

[bb0095] Gelman A., Rubin (1992). Inference from iterative simulation using multiple sequences. Stat. Sci..

[bb0100] Hu X., Song S., Ye F., Liu L. (2006). High-performance liquid chromatographic determination of urinary trans, trans-muconic acid excreted by workers occupationally exposed to benzene. Biomedical & Environmental Sciences.

[bb0110] International Agency for Research on Cancer (IARC) (1989). Occupational exposures in petroleum refining: crude oil and major petroleum fuels. IARC Monographs.

[bb0115] Jiřík V., Machaczka O., Miturová H., Tomášek I., Šlachtová H., Janoutová J., Velická H., Janout V. (2016). Air pollution and potential health risk in Ostrava region – a review. Cent. Eur. J. Public Health.

[bb0120] Kang S., Lee M., Kim T., Lee J., Ahn Y. (2005). Occupational exposure to benzene in South Korea. Chem. Biol. Interact..

[bb0125] Kirkeleit J., Riise T., Bråtveit M., Moen B. (2008). Increased risk of acute myelogenous leukaemia and multiple myeloma in a historical cohort of upstream petroleum workers exposed to crude oil. Cancer Causes Control.

[bb0130] Knox E. (1994). Leukaemia clusters in childhood: geographical analysis in Britain. J. Epidemiol. Community Health.

[bb0135] Lan Q., Zhang L., Li G., Vermeulen R., Weinberg R., Dosemeci M., Rappaport S., Shen M., Alter B., Wu Y., Kopp W., Waidyanatha S., Rabkin C., Guo W., Chanock S., Hayes R.B., Linet M., Kim S., Yin S., Rothman N., Smith M. (2004). Hematotoxicity in workers exposed to low levels of benzene. Science..

[bb0140] Lin C., Hung H., Christiani D., Forastiere F., Lin R. (2017). Lung cancer mortality of residents living near petrochemical industrial complexes: a meta-analysis. Environ. Health.

[bb9000] Lin C., Hsu Y., Christiani D., Hung H., Lin R. (2018). Risks and burden of lung cancer incidence for residential petrochemical industrial complexes: a meta-analysis and application. Environ. Int..

[bb0155] Molle W. (1984). Oil refineries and petrochemical industries in Europe. Geojournal.

[bb0160] Morello-Frosch R., Pastor M., Porras C., Sadd J. (2002). Environmental justice and regional inequality in Southern California: implications for future research. Environ. Health Perspect..

[bb0165] Nagelkerke N. (1991). A note on a general definition of the coefficient of determination. Biometrika.

[bb0170] Neidell M & Lavaine E (2012). Morbidity and sulfur dioxide: Evidence from French strikes at oil refineries. *Paris School of Economics*. Available via: parisschoolofeconomics.eu/IMG/pdf/january2012.pdf.

[bb0175] Pascal L., Pascal M., Stempfelet M., Goria S., Declercq C. (2013). Ecological study on hospitalizations for cancer, cardiovascular, and respiratory diseases in the industrial area of Etang-de-Berre in the south of France. *Journal of Environmental and Public Health*. Article ID.

[bb0180] Pasetto R., Zona A., Pirastu R., Cernigliaro A., Dardanoni G., Addario S.P., Scondotto S., Comba P. (2012). Mortality and morbidity study of petrochemical employees in a polluted site. Environ. Health.

[bb0185] Paxton M., Chinchilli V., Brett S., Rodricks J. (1994). Leukemia risk associated with benzene exposure in the pliofilm cohort: I. Mortality update and exposure distribution. Risk Anal..

[bb0190] Pitard A., Zeghnoun A., Courseaux A., Lamberty J., Delmas V., Fossard J.L., Villet H. (2004). Short-term associations between air pollution and respiratory drug sales. Environ. Res..

[bb0195] Plummer M. (2003). JAGS: A program for analysis of Bayesian graphical models using Gibbs sampling. *The third international workshop on 'Distributed Statistical Computing' (DSC)*, University of Vienna, 20^th^-23^rd^ March 2003.

[bb0200] Ramis R., Vidal E., García-Pérez J., Lope V., Aragonés N., Pérez-Gómez B., Pollán M., López-Abente G. (2009). Study of non-Hodgkin's lymphoma mortality associated with industrial pollution in Spain, using Poisson models. BMC Public Health.

[bb0205] Rusconi F., Catelan D., Accetta G., Peluso M., Pistelli R., Barbone F., Di Felice E., Munnia A., Murgia P., Paladini L., Serci A., Biggeri A. (2011). Asthma symptoms, lung function, and markers of oxidative stress and inflammation in children exposed to oil refinery pollution. J. Asthma.

[bb0215] Sans S., Elliott P., Kleinschmidt I., Shaddick G., Pattenden S., Walls P., Grundy C., Dolk H. (1995). Cancer incidence and mortality near the Baglan Bay petrochemical works, South Wales. Occup. Environ. Med..

[bb9010] Simonsen N., Scribner R., Su J., Williams D., Luckett B., Yang T., Fontham E. (2010). Environmental exposure to emissions from petrochemical sites and lung cancer: the Lower Mississippi Interagency Cancer Study. J. Environ. Public Health.

[bb9005] Smargiassi A., Kosatsky T., Hicks J., Plante C., Armstrong B., Villeneuve P., Goudreau S. (2009). Risk of asthmatic episodes in children exposed to sulfur dioxide stack emissions from a refinery point source in Montreal, Canada. Environ. Health Perspect..

[bb0220] Sram R., Dostal M., Libalova H., Rossner P., Rossnerova A., Svecova V., Topinka J., Bartonova A. (2013). The European hot spot of B[a]P and PM2.5 exposure—the Ostrava region, Czech Republic: health research results. ISRN Public Health.

[bb0230] Trafimow D., Marks M. (2015). Editorial. Basic Appl. Soc. Psychol..

[bb0235] Tukiendorf A. (2004). Comparison of lung cancer incidence with air pollution level. Case study – Kędzierzyn-Koźle. Chemia i Inżynieria Ekologiczna.

[bb0240] United States Environmental Protection Agency (US EPA) (2012). Chemical-specific reference values for benzene. National Center for Environmental Assessment. EPA/600/R-12/047F1.

[bb0245] Velická H., Puklová V., Keder J., Brabec M., Malý M., Bobák M., Kotlík B., Jiřík V., Janout V., Kazmarová H. (2015). Asthma exacerbations and symptom variability in children due to short-term ambient air pollution changes in Ostrava, Czech Republic. Cent. Eur. J. Public Health.

[bb0250] Walker G. (2012). Environmental Justice.

[bb0255] Widziewicz K., Rogula-Kozłowska W., Majewski G. (2017). Lung cancer risk associated with exposure to benzo(a)pyrene in polish agglomerations, cities, and other areas. International Journal of Environmental Research.

[bb0260] Wilkinson P., Thakrar B., Walls P., Landon M., Falconer S., Grundy C., Elliott P. (1999). Lymphohaematopoietic malignancy around all industrial complexes that include major oil refineries in Great Britain. Occup. Environ. Med..

[bb0265] World Health Organisation (WHO) (2010). WHO Guidelines for Indoor Air Quality: Selected Pollutants.

[bb0270] World Health Organisation (WHO) (2010). Exposure to Benzene: A Major Public Health Concern.

[bb0275] World Health Organisation (WHO) (2014). Human Health in Areas With Industrial Contamination.

[bb0280] Zeghnoun A, Czernichow P, Beaudeau P, Hautemanière A, Froment L, LE Tertre A, & Quénel P (2010). Short-term effects of air pollution on mortality in the cities of Rouen and Le Havre, France, 1990-1995. Arch. Environ. Health, 56:4, pp.327–335. 10.1080/00039890109604464 11572276

